# Knowledge, Lifestyle, and Attitudes Toward Nutritional Assessment and Counseling Among Physiotherapists in Saudi Arabia: Implications for Healthcare Quality and Interdisciplinary Practice—A Cross-Sectional Study Across Multiple Regions of Saudi Arabia

**DOI:** 10.3390/healthcare14142101

**Published:** 2026-07-14

**Authors:** Mohamed M. Ahmed, Azza A. Al Areefy, Rama M. Chandika, Ramzi Abdu Alajam, Alanoud Huraysi, Marim Ali M. Slimani, Bsmah H. Alfaifi, Huda M. Mobarki, Laila Shamakhi, Ehab Y. Elbendary, Wafaa Mahmoud Amin

**Affiliations:** 1 Department of Physical Therapy, College of Nursing and Health Sciences, Jazan University, Jazan 82817, Saudi Arabia; mmahmed@jazanu.edu.sa (M.M.A.); ralajam@jazanu.edu.sa (R.A.A.); ajhureysi@jazanu.edu.sa (A.H.); mariamali@jazanu.edu.sa (M.A.M.S.); bhjabor@jazanu.edu.sa (B.H.A.); 2 Department of Basic Science in Physical Therapy, Faculty of Physical Therapy, Beni Suef University, Beni Suef 62511, Egypt; 3 Clinical Nutrition Department, College of Nursing and Health Sciences, Jazan University, Jazan 82817, Saudi Arabia; aalareefy@jazanu.edu.sa (A.A.A.A.); rchandika@jazanu.edu.sa (R.M.C.); hmobarki@jazanu.edu.sa (H.M.M.); shamakhi.laila@outlook.com (L.S.); eelbendary@jazanu.edu.sa (E.Y.E.); 4 Department of Nutrition and Food Science, Faculty of Home Economics, Helwan University, Helwan 11711, Egypt; 5 Specialized Medical Hospital, Faculty of Medicine, Mansoura University, Mansoura 35516, Egypt; 6 Department of Basic Science for Physical Therapy, Faculty of Physical Therapy, Cairo University, Giza 12613, Egypt

**Keywords:** physical therapy, nutritional assessment, nutrition integration, healthcare quality, interdisciplinary practice, nutrition education, rehabilitation, Saudi Arabia

## Abstract

**Background/Objectives**: Nutrition is essential in healthcare and rehabilitation, although the readiness of physiotherapists in Saudi Arabia to integrate nutritional assessment and counseling remains ambiguous. This study aimed to assess their knowledge of nutrition, lifestyle choices, and attitudes towards incorporating nutrition into practice. **Methods**: This cross-sectional study included 500 licensed physiotherapists practicing across Saudi Arabia and used a culturally adapted questionnaire derived from previously validated instruments. The survey assessed background characteristics, nutrition knowledge, nutritional lifestyle, and attitudes toward integrating nutrition into physical therapy. Descriptive statistics, analysis of variance, Pearson correlation, and multiple linear regression analyses were performed. **Results**: Participants demonstrated moderate nutrition knowledge (59.68 ± 17.25) and favorable nutritional lifestyles (20.67 ± 3.79), with positive attitudes toward integrating nutritional assessment and counseling into their practice (13.63 ± 4.64). A significant positive correlation was observed between nutritional lifestyle scores and nutrition integration scores (r = 0.29, *p* < 0.01). Because lower nutritional lifestyle scores indicate healthier lifestyles and lower integration scores reflect greater integration, participants with healthier nutritional lifestyles tend to show greater nutrition integration. Nutrition knowledge scores were negatively correlated with integration scores (r = −0.37, *p* < 0.01). Multiple linear regression analysis identified professional seniority, nutritional lifestyle, and nutrition knowledge as significant predictors of nutrition integration score, explaining 22.8% of the variance (F = 29.24, R^2^ = 0.22, *p* = 0.001). **Conclusions**: Saudi physiotherapists showed positive attitudes toward integrating nutritional assessment and counseling into the practice, but their nutrition knowledge was moderate, with knowledge gaps. Nutrition integration correlated significantly with nutritional lifestyle scores, nutrition knowledge, and professional seniority. The findings indicate the need to improve nutrition-related competencies and interdisciplinary collaboration in rehabilitation practice.

## 1. Introduction

Noncommunicable diseases (NCDs) are a leading cause of disability and healthcare use. Two major modifiable drivers, unhealthy diet and insufficient physical activity, often cluster in the same individuals [1]. In Saudi Arabia, national surveillance reports for adolescents and adults aged 15 years and older indicate a high prevalence of excess weight, with 23.1% classified as obese and 45.1% as overweight [2]. Recent analyses also describe a measurable health and economic burden of insufficient physical activity in Saudi Arabia, supporting prevention strategies that combine movement and nutrition within routine care [3].

These patterns strengthen the rationale for coordinated lifestyle-focused prevention across healthcare disciplines, including rehabilitation services where long-term function and self-management are central goals. Physical therapy is increasingly positioned not only as impairment-focused rehabilitation but also as a contributor to population health and chronic disease self-management through its roles in prompting health, optimizing physical function, and providing interdisciplinary care [4,5,6].

Nutritional status and diet quality can influence rehabilitation outcomes by affecting muscle mass, strength, inflammation, metabolic health, and exercise tolerance. Rehabilitation nutrition perspectives highlight that combined nutrition and exercise interventions can improve aspects of physical performance in conditions such as sarcopenia [7,8,9]. A position paper focused on nutrition and physical therapy describes a bidirectional relationship: nutritional management can enhance the effectiveness of physical therapy, and physical therapy can enhance the effectiveness of nutritional management [10]. From an implementation standpoint, this requires clinicians to recognize nutrition-related risks that may limit progress (e.g., unintentional weight loss, inadequate protein intake, or low diet quality) and to integrate screening and referral pathways into routine care [7].

The professional guidance on the established role of physiotherapists in nutrition-related care, particularly in rehabilitation, is to identify nutritional risk factors, perform basic screening, promote healthy lifestyle principles, and refer for specialized dietary management [10,11]. The term “nutritionist” is not standardized in terms of qualifications. Still, registered dietitians have formal clinical training in medical nutrition therapy [7,12] and play an important role in interdisciplinary collaboration and safe nutrition-related rehabilitation care [10,11]. Despite this support, international evidence indicates that integration in practice remains inconsistent and is often constrained by limited training, time pressures, and uncertainty about professional boundaries. Surveys of physiotherapists report positive attitudes but variable adherence to routine [13,14,15].

Physical therapy education in Saudi Arabia is typically delivered as a 5- to 6-year bachelor’s program that includes a preparatory year and a supervised internship, with notable variation in the number of credit units and course structures across universities [16]. Postgraduate education was introduced in Saudi Arabia in 2000, with major tracks including pediatrics, geriatrics, orthopedics, neurology, and cardiothoracic physical therapy, and early models emphasized research training [16]. Although undergraduate curricula are comprehensive in foundational biomedical sciences and core physical therapy specialty content, nutrition-related competencies are not consistently articulated as a discrete domain in physical therapy program descriptions. This may contribute to variability in graduates’ confidence and readiness to conduct nutrition screening, provide brief counseling, and establish appropriate referral pathways [12,17].

At the postgraduate level, the Saudi Commission for Health Specialties (SCFHS) has adopted competency-based curricula for advanced clinical training programs such as the Saudi Board of Musculoskeletal Physical Therapy, which emphasizes specialty clinical reasoning, therapeutic exercise, manual therapy, and patient education [18]. While these curricula position physical therapists as health advocates and educators, nutrition-specific learning outcomes are not explicit, suggesting that nutrition integration depends on local training opportunities and individual initiative [12]. Some Saudi physical therapy programs incorporate prevention and health promotion coursework (e.g., health promotion and prevention practice modules) that can provide a conceptual foundation for lifestyle counseling [18,19,20,21]. However, the extent to which nutrition assessment tools and counseling skills are formally taught, particularly in rehabilitation settings, remains unclear. This curricular context strengthens the justification for examining practicing physical therapists’ attitudes toward integrating nutrition and the factors associated with more favorable attitudes.

Studies have shown that malnutrition and improper nutritional management are closely associated with poor rehabilitation outcomes and impaired physical function [8]. Whereas incorporating nutritional assessment and counseling into physical rehabilitation can enhance patient-centered care and healthcare quality [8,9]. Therefore, this nationwide cross-sectional study aimed to assess physical therapists’ attitudes toward integrating nutritional assessment and counseling into clinical practice in Saudi Arabia, to support education planning and safe interprofessional implementation, and to emphasize their implications for healthcare quality and interdisciplinary practice.

## 2. Materials and Methods

### 2.1. Study Design

This study employed a cross-sectional design to examine nutrition knowledge, nutritional lifestyle, and attitudes toward integrating nutritional assessment and counseling within physical therapy practice among licensed physiotherapists in Saudi Arabia.

### 2.2. Ethical Considerations

Ethical approval was obtained from the Local Committee for Research Ethics at Jazan University (HAPO-10-Z-001), reference number REC-46/06/1324. All participants provided informed consent before participation.

#### 2.2.1. Setting and Sample Size Calculation

The target population comprised licensed physical therapists practicing across all administrative regions of the Kingdom of Saudi Arabia. Convenience sampling was used to recruit physiotherapists. Recruitment aimed to achieve broad geographical coverage across the Central, Eastern, Western, Northern, and Southern regions of Saudi Arabia. A prevalence of 55% was reported in a Saudi Arabian study of physiotherapists assessing disease management-related knowledge [21]. Using a 5% significance level and a 5% margin of error, the sample size was calculated to be 380. Accounting for a 20% nonresponse rate of 456, the final sample size was adjusted to 500 physiotherapists.

#### 2.2.2. Sampling Frame

A total of 600 physiotherapists were recruited; 518 initially responded to participate in the study, yielding a response rate of 86%. After eligibility screening, 18 respondents were excluded, resulting in a final analytical sample of 500 physiotherapists.

#### 2.2.3. Eligibility Criteria

Participants were excluded if they were physical therapy interns, assistants, or students (*n* = 10); practicing outside Saudi Arabia (*n* = 1); primarily engaged in academic roles without active clinical practice (*n* = 4); or held a diploma in nutrition (*n* = 3). Inclusion criteria were licensed physical therapists practicing in Saudi Arabia, at least 1 year of clinical experience, and willingness to provide informed consent.

### 2.3. Geographical Distribution

**Figure 1:**
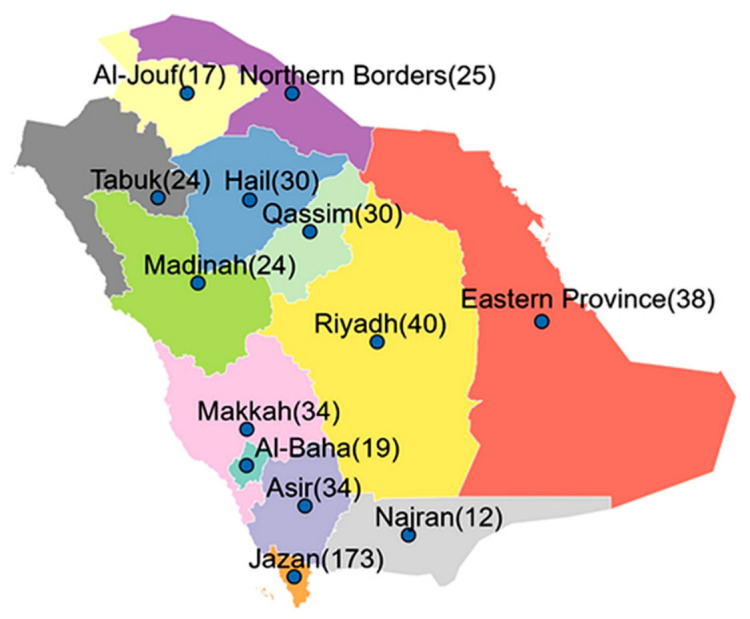
Geographical distribution of participating physiotherapists across regions of Saudi Arabia.

Participants were recruited from across Saudi Arabia. The geographical distribution of the sample is illustrated in Figure 1, demonstrating representation from Jazan, Riyadh, Makkah, Eastern Province, Qassim, Hail, Madinah, Tabuk, Northern Borders, Al-Jouf, Asir, Al-Baha, and Najran.

#### 2.3.1. Survey Administration and Recruitment

The questionnaire was administered as an online survey. The survey link was distributed through various healthcare facilities across Saudi Arabia, including hospitals, outpatient clinics, and rehabilitation centers. In addition, recruitment was supported through professional physical therapy conferences and events held in Saudi Arabia, such as the Saudi Physical Therapy Association (SPTA) 2024 conference. This multi-channel distribution strategy was adopted to enhance national reach and facilitate participation from physical therapists practicing in diverse clinical settings across the Kingdom.

#### 2.3.2. Questionnaire Development and Cultural Adaptation

The data collection instrument was a structured, self-administered questionnaire previously used in published research assessing the integration of nutritional assessment and counseling within physical therapy practice. The original questionnaire demonstrated acceptable validity and reliability [14,15]. For the present study, the questionnaire was culturally adapted to the Saudi context by modifying terminology, examples, and dietary references to align with Saudi Ministry of Health guidelines, cultural traditions, and professional regulations, while preserving the original conceptual framework and scale structure. After translation and cultural adaptation, the questionnaire was revised by a multidisciplinary team of academic experts in physical therapy and clinical nutrition for content relevance, clarity, and cultural appropriateness, with minor wording adjustments made before pilot testing.

#### 2.3.3. Questionnaire Structure

The final questionnaire consisted of four sections: background characteristics; nutrition knowledge, assessed using a 17-item scale with 5.88 scores for each item [15]; nutritional lifestyle, measured using a 7-item Likert scale; and integration of nutritional assessment and counseling in physical therapy practice, assessed using a 5-item Likert scale. Lower scores indicated healthier nutritional lifestyles and greater integration of nutrition. The questionnaire was translated into Arabic through a standardized forward -backward translation process performed by independent bilingual translators. A pilot study was conducted with 25 physiotherapists who were asked to provide feedback on the clarity, wording, comprehension, and cultural appropriateness of questionnaire items. Minor linguistic adjustments were subsequently made in response to the feedback. Internal consistency was acceptable (Cronbach’s α = 0.76 for nutritional lifestyle; α = 0.82 for nutritional integration).

#### 2.3.4. Statistical Analysis

Data was analyzed using IBM SPSS Statistics for Windows, version 27.0. Descriptive statistics were presented as frequencies, percentages, range, means, and standard deviations. To determine variations in nutrition integration in physical therapy practice, Analysis of variance (ANOVA) was applied as a function of professional seniority level. Bivariate Pearson correlation was used to assess the association between nutrition integration and physiotherapists’ background variables, including age (years), BMI, education level, nutrition lifestyle, and nutrition knowledge. Multiple linear regression was used to identify predictors: professional seniority, nutritional lifestyle, and nutrition knowledge of nutrition integration in physical therapy practice, with age included as a potential confounding variable. A geographical plot of Saudi Arabia was constructed using R software (version 4.1.2). A two-tailed *p*-value < 0.05 was considered statistically significant.

## 3. Results

### 3.1. Participant Characteristics

A total of 500 licensed physiotherapists practicing in Saudi Arabia were included in the final analysis. Participants had a mean age of 27.6 ± 6.32 years and a mean body mass index (BMI) of 23.76 ± 5.39 kg/m^2^.

Regarding professional seniority, most participants reported 1–2 years of clinical experience (308, 61.6%), followed by 3–12 years (146, 29.2%) and ≥13 years (46, 9.2%). Most physiotherapists held a bachelor’s degree (419, 83.8%), while smaller proportions held a diploma (24, 4.8%), a master’s degree (39, 7.8%), or a PhD (18, 3.6%).

Regarding undergraduate education, 34.6% of participants reported disagreeing or strongly disagreeing that they had learned when to refer a patient for dietary advice during their undergraduate training. Additionally, a substantial proportion of participants reported being unsure or lacking sufficient knowledge to perform an initial nutritional review as part of patient assessment. Detailed background characteristics are presented in Table 1.

### 3.2. Geographical Distribution of Participants

The participating physiotherapists were recruited from across the Kingdom of Saudi Arabia, reflecting broad geographical coverage. As shown in Figure 1, respondents were distributed across the Central, Western, Eastern, Northern, and Southern regions, with representation from major administrative areas including Riyadh, Makkah, Eastern Province, Madinah, Qassim, Jazan, Asir, Al-Baha, Najran, Tabuk, Hail, Northern Borders, and Al-Jouf.

### 3.3. Nutrition Knowledge

Nutrition knowledge was assessed using a 17-item questionnaire. The mean total nutrition knowledge score among physiotherapists was 59.68 ± 17.25 (out of 100). Physiotherapists demonstrated relatively strong knowledge in selected areas. For example, 53.8% correctly identified the combination of vitamins and minerals essential for maintaining normal bone mass, and 57.0% correctly recognized zinc as an important mineral for immune system function. In contrast, knowledge gaps were evident in areas related to national dietary recommendations and clinical referral thresholds. Approximately 50.6% of participants were unaware that the Ministry of Health’s food recommendations prioritize unprocessed, plant-based foods, and 47.8% were unable to identify the serum albumin level indicative of suspected malnutrition that requires referral. The distribution of correct responses for each knowledge item is summarized in Supplementary Table S1.

### 3.4. Nutritional Lifestyle

Nutritional lifestyle was evaluated using a 7-item scale, which demonstrated acceptable internal consistency (Cronbach’s α = 0.76). Lower scores indicate healthier nutritional lifestyles. The mean total nutritional lifestyle score was 20.67 ± 3.79 (range: 7–35), reflecting an overall moderate-to-favorable nutritional lifestyle among participants.

Participants reported relatively infrequent fast-food consumption (2.66 ± 1.21) and moderate self-ratings of their overall nutritional lifestyle (2.67 ± 1.10). Preferences for non-processed foods (2.90 ± 1.27) and maintaining a healthy body weight (2.83 ± 1.37) were also reported. Consumption of animal protein more than three times per week showed the highest mean score (3.39 ± 1.36). Item-level results are presented in Table 2.

### 3.5. Integration of Nutritional Assessment and Counseling in Physical Therapy Practice

Attitudes toward integrating nutritional assessment and counseling into physical therapy practice were assessed using a 5-item scale, which demonstrated strong internal consistency (Cronbach’s α = 0.82). Lower scores indicated higher levels of integration. The mean total integration score was 13.63 ± 4.64 (range: 5–25), indicating a generally favorable attitude toward nutrition integration. Participants moderately agreed that an introductory nutrition course should be mandatory in undergraduate physical therapy curricula (2.79 ± 1.29) and that such courses should be delivered by registered clinical nutritionists (2.81 ± 1.23). Agreement was also observed regarding the importance of physiotherapists’ understanding of nutritional principles and the incorporation of nutritional considerations into rehabilitation processes. Detailed results are shown in Table 3.

### 3.6. Comparison by Professional Seniority

The mean total score on the Nutrition Integration in Physical Therapy Practice scale was comparable across professional seniority levels. Physiotherapists with 1–2 years of experience had a mean score of 13.56 (SD = 4.85), those with 3–12 years had 13.74 (SD = 4.28), and those with ≥13 years had 13.72 (SD = 4.44). The overall mean score was 13.63 (SD = 4.64), as presented in Table 4.

### 3.7. Correlation Analysis

Bivariate correlation analysis demonstrated significant inverse relationships between total nutrition integration scores and age (r = −0.21, *p* < 0.01), BMI (r = −0.11, *p* < 0.05), and education level (r = −0.06, *p* > 0.05). In contrast, a positive correlation was observed between total integration scores and nutritional lifestyle scores (r = 0.29, *p* < 0.01); lower scores indicate greater integration of nutrition and healthier nutritional lifestyles. This indicates that participants with healthier nutritional lifestyles (lower nutritional lifestyle scores) exhibited greater nutrition integration (lower integration scores). Also, a negative correlation was found between integration scores and nutrition knowledge scores (r = −0.37, *p* < 0.01), indicating that higher nutrition knowledge was associated with greater nutrition integration. These findings are summarized in Table 5.

### 3.8. Predictors of Nutrition Integration in Physical Therapy Practice

Multiple linear regression analysis was conducted to identify predictors of nutrition integration scores within physical therapy practice. The model was statistically significant (F (5, 494) = 29.24, *p* < 0.01) and explained 22.84% of the variance in nutrition integration scores (R^2^ = 0.228). Professional seniority, nutritional lifestyle, and nutrition knowledge emerged as significant predictors. Compared with physiotherapists with ≥13 years of experience, those with 1–2 years of experience demonstrated significantly lower integration scores (β = −0.21, *p* < 0.05). Higher nutritional lifestyle scores, which indicate less favorable nutritional lifestyles, were associated with higher nutrition integration scores, which indicate a lower level of nutrition integration (β = 0.21, *p* < 0.01). In contrast, higher nutrition knowledge scores were associated with lower nutrition integration scores, indicating greater nutrition integration. (β = −0.29, *p* < 0.01). Regression results are presented in Table 6.

## 4. Discussion

This cross-sectional study examined nutrition knowledge, nutritional lifestyle, and attitudes toward integrating nutritional assessment and counseling within physical therapy practice among licensed physiotherapists in Saudi Arabia. The findings provide insight into physiotherapists’ knowledge of nutrition, lifestyle behaviors, and attitudes toward integrating nutrition into rehabilitation practice in Saudi Arabia.

The results indicate that Saudi physiotherapists demonstrated moderate nutritional knowledge, generally favorable nutritional lifestyles, and positive attitudes toward integrating nutrition. However, the inverse association between nutrition knowledge and nutrition integration scores suggests that higher nutrition knowledge among physiotherapists is associated with greater nutrition integration in rehabilitation practice. This finding suggests that nutrition knowledge may facilitate the integration of nutritional considerations into physiotherapy practice. Previous research has shown that greater nutrition knowledge and confidence lead to a stronger willingness to address nutrition-related issues in clinical care [14,15,16]. Furthermore, recent evidence indicates that enhanced nutrition knowledge correlates with healthier dietary behaviors and improved adherence to healthy dietary patterns, suggesting that nutrition knowledge may be associated with both personal health practices and professional health-related behaviors [22].

Although overall nutrition knowledge was moderate, important gaps were identified in areas relevant to clinical decision-making, including national dietary recommendations and biochemical thresholds indicating malnutrition risk. This aligns with prior international studies that highlight variability and inconsistency in nutrition-related education and its implementation by physiotherapists [13,14,15].

A positive association was observed between nutritional lifestyle scores and nutrition integration scores. Given the instruments’ scoring structure, in which lower scores indicate healthier nutritional lifestyles and greater nutrition integration, participants with healthier nutritional lifestyles (lower nutritional lifestyle scores) tended to demonstrate greater nutrition integration (lower integration scores). These findings suggest a relationship between personal lifestyle behaviors and nutrition-related attitudes and practices within rehabilitation settings. Similar associations between healthcare professionals’ personal health behaviors and their engagement in lifestyle-related counseling have been reported previously [16,23]. However, the cross-sectional design of the study limits the ability to draw causal conclusions.

Professional seniority was not significantly associated with nutrition integration scores in the unadjusted ANOVA analysis. However, in the multivariable regression model, physiotherapists with 1–2 years of experience demonstrated significantly lower integration scores than those with ≥13 years of experience after adjustment for age and other covariates. This finding suggests that differences in nutrition integration across career stages may become apparent only after accounting for potential confounding factors and necessitate further investigation.

The observed heterogeneity in nutrition integration may partially indicate variability in educational background and clinical experience among physiotherapists in Saudi Arabia. While participants exhibited positive attitudes toward incorporating nutrition, the existing knowledge gap and variable application indicate that current training may not uniformly prepare physiotherapists to address nutrition-related challenges in rehabilitation contexts [13,14,15,18]. The integration of nutrition into physiotherapy should be considered within professional boundaries. Physiotherapists can identify nutrition-related risk factors, conduct basic screenings, and promote healthy lifestyle principles relevant to rehabilitation. However, comprehensive dietary management of complex nutritional issues falls under the expertise of registered dietitians. The findings underscore the importance of interdisciplinary collaboration and referral pathways for safe, patient-centered nutrition care in rehabilitation settings [7,10,23]. Based on our findings, patients who require specialized nutritional assessment or individualized dietary management may benefit from clear interdisciplinary referral pathways that involve registered dietitians.

The strengths of this study include a large sample recruited from multiple geographical regions of Saudi Arabia and the use of previously validated instruments adapted to the Saudi context.

Several limitations should be considered. First, the cross-sectional design limits causal inference. Second, the self-reported data may have been subject to recall, social desirability, and response biases. Third, the use of an online survey for data collection may have introduced selection bias, as physiotherapists who are more digitally engaged or professionally active may have been more likely to participate. Finally, although participants were recruited from various regions in Saudi Arabia, convenience sampling might have led to over- or underrepresentation of specific geographical areas or professional subgroups, thereby limiting the generalizability of the findings to all physiotherapists in the country. Furthermore, formal cognitive interviewing and factor-analytic validation of the culturally adapted questionnaire were not conducted. Although expert review and pilot testing supported the content’s relevance and comprehensibility, further psychometric evaluation in the Saudi context is needed in future research.

Future interventional and qualitative studies are needed to investigate further the associations between nutrition-related competencies and decision-making, rehabilitation practice, and interdisciplinary collaboration within physical therapy settings.

## 5. Conclusions

Saudi physiotherapists have a positive attitude towards the inclusion of nutritional assessment in practice, moderate nutritional knowledge, and generally favorable nutritional lifestyles. Nutrition integration was significantly associated with nutritional lifestyle scores, nutrition knowledge, age, and professional seniority. Variation in nutrition integration and knowledge was observed across professional seniority groups, particularly among early-career physiotherapists. These findings support the importance of nutrition-related competencies within physiotherapy practice. The findings underscore the importance of interdisciplinary collaboration and appropriate referral pathways that involve registered dietitians when individualized nutritional management is indicated.

## Figures and Tables

**Table 1:** Background characteristics.

Characteristics	Categories	Physiotherapists *N* = 500 (%)
Age, mean (SD)		27.6 (6.32)
BMI, mean (SD)		23.76 (5.39)
Professional seniority	1–2 years	308 (61.60)
	3–12 years	146 (29.20)
	≥13 years	46 (9.20)
Academic degrees	Diploma	24 (4.80)
	Bachelor	419 (83.80)
	Master	39 (7.80)
	PhD	18 (3.60)
During my undergraduate degree, I learned when to refer a patient for dietary advice	S A	36 (7.20)
	Agree	194 (38.80)
	Unsure	96 (19.20)
	Disagree	107 (21.40)
	S D	67 (13.40)
I have sufficient knowledge to perform an initial nutritional review to assess the patient’s nutritional status.	S A	26 (5.20)
	Agree	205 (41.00)
	Unsure	125 (25.00)
	Disagree	84 (16.80)
	S D	60 (12.00)

SD: Standard deviation; BMI: Body Mass Index; S A: Strongly Agree; S D: Strongly Disagree.

**Table 2:** Nutritional Lifestyle.

Variable	Mean (SD)
Evaluation of Nutrition Lifestyle (The lower the score, the higher the nutritional lifestyle of the participants) [Range: 1–5]	
How many times a week do you eat fast food outside the house or order fast food?	2.66 (1.21)
How would you rate your nutritional lifestyle?	2.67 (1.10)
I tend to minimize the consumption of processed/ultra-processed foods and prefer non-processed foods.	2.90 (1.27)
As a part of my food selection process, I tend to look at its ingredients, nutritional components, and whether it carries a Ministry of Health red/green label.	3.06 (1.15)
I tend to prefer plant-based foods in my daily diet, making sure to diversify with legumes, fruits, vegetables, nuts, and vegetable oils.	3.16 (1.19)
I consume animal protein in the form of meat/chicken more than 3 times a week	3.39 (1.36)
I maintain a normal body weight to reduce the risk of developing chronic diseases in the present/future	2.83 (1.37)
Nutrition Lifestyle mean score [Range: 7–35]	20.67 (3.79)

**Table 3:** Integration of nutritional assessment and counseling in physical therapy treatment.

Variable	Mean (SD)
Evaluation of nutrition integration in the physical therapy practice (The lower the average score, the higher levels of integration) [Range: 1–5]	
An introductory course on nutrition should be mandatory in the curriculum for a bachelor’s degree in physical therapy	2.79 (1.29)
A registered dietitian should give an introductory nutrition course.	2.81 (1.23)
Physiotherapists should have a good understanding of nutritional principles/recommendations	2.63 (1.20)
The rehabilitation process within the framework of physiotherapy must also include personal education on a balanced diet by the physiotherapist	2.75 (1.16)
Do you think physiotherapy should include reference to the nutritional status of the patient (by referring to a professional or by the physiotherapist)?	2.66 (1.18)
Nutrition integration in the physical therapy practices means a score [Range: 5–25]	13.63 (4.64)

**Table 4:** Comparison of Total Nutrition Integration Scores Across Professional Seniority Groups.

Total Nutrition Integration in Physical Therapy Practice Scale Score				
Measure		Mean (SD)	^§^ Test Statistic	*p*-Value
Professional seniority	1–2 years	13.56 (4.85)	F (2, 497) = 0.08 and *p* = 0.92	
	3–12 years	13.74 (4.28)		
	≥13 years	13.72 (4.44)		
	Overall	13.63 (4.64)		

^§^: Analysis of variance, *p* < 0.05 was considered statistically significant.

**Table 5:** Pearson Correlation Analysis Between Nutrition Integration Scores and Participant Characteristics.

Total Nutrition Integration in Physical Therapy Practice Scale Score		
Variable	Mean (SD)	r (*p*-Value)
Age (years)	27.6 (6.32)	−0.21 **
BMI	23.76 (5.39)	−0.11 *
Education level	--	−0.06
Total nutritional lifestyle score	20.67 (3.79)	0.29 **
Total nutrition knowledge score	59.68 (17.25)	−0.37 **

r: Pearson correlation coefficient. Nutrition integration scores represent total scores, with lower values indicating greater integration. Nutritional lifestyle scores are total scores; lower values indicate healthier nutritional lifestyles. *: significant (*p* < 0.05); **: highly significant (*p* < 0.01).

**Table 6:** Multiple Linear Regression Analysis Identifying Predictors of Nutrition Integration in Physical Therapy Practice.

Total Nutrition Integration in Physical Therapy Practice Scale Score				
Variable	Parameter Estimate	Standard Error	Standardized Estimate	Value of ‘t’ (*p*-Value)
Professional seniority—1–2 years vs. over 13 years	−2.04	0.74	−0.21	−2.76 *
Professional seniority—3–12 years vs. over 13 years	−0.85	0.72	−0.08	−1.18
Age	−0.19	0.03	−0.26	−5.57 **
Total nutritional lifestyle score	0.26	0.05	0.21	5.26 **
Total nutrition knowledge score	−0.08	0.01	−0.29	−7.15 **
F (5, 494) = 29.24 **, R^2^: 22.84%				

Standardized Estimate represents the standardized regression coefficient (β), R^2^ = coefficient of determination/Reference category for professional seniority: ≥13 years, *: significant (*p* < 0.05), **: highly significant (*p* < 0.01).

## Data Availability

The data presented in this study are available on request from the corresponding author due to ethical and confidentiality constraints.

## Supplementary Materials

The following supporting information can be downloaded at: https://www.mdpi.com/article/10.3390/healthcare14142101/s1, Table S1: Nutrition Knowledge Level among physiotherapists in Saudi Arabia.
