# Supplementary material for: Knowledge, Lifestyle, and Attitudes Toward Nutritional Assessment and Counseling Among Physiotherapists in Saudi Arabia: Implications for Healthcare Quality and Interdisciplinary Practice—A Cross-Sectional Study Across Multiple Regions of Saudi Arabia
Source: Healthcare (Basel). 2026 Jul 14;14(14):2101. doi: 10.3390/healthcare14142101 (PMC13410139; doi:10.3390/healthcare14142101)
Supplement: Supplementary file 1 [file healthcare-14-02101-s001.zip › healthcare-4272107-supplementary.pdf]

Supplementary Table S1. Nutrition Knowledge Level among physiotherapists in Saudi Arabia.

Item		Physiotherapists N = 500(%)
It is recommended to eat lean chicken/turkey every day	True	165 (33.00)
	False	186 (37.20)
	Don't know	149 (29.80)
It is recommended to minimize the consumption of red meat/beef to consume no more than 300 g per week	True	250 (50.00)
	False	114 (22.80)
	Don't know	136 (27.20)
Industrial foods (ketchup, snacks, processed meat) are allowed for consumption in the amount of 2–3 portions per week	True	203 (40.60)
	False	118 (23.60)
	Don't know	179 (35.80)
The food arrangement recommended by the Ministry of Health is based on consuming unprocessed (natural) foods, primarily plant based.	True	247 (49.40)
	False	115 (23.00)
	Don't know	138 (27.60)
The recommendation for proper nutrition, according to the Ministry of Health, is based on the recommended frequency of consumption	True	264 (52.80)
	False	82 (16.40)
	Don't know	154 (30.80)
The recommendation for proper nutrition, according to the Ministry of Health, does not refer to the degree of processing of the food	True	172 (34.40)
	False	171 (34.20)
	Don't know	157 (31.40)
What is the role of calcium in the body?	An essential mineral for muscle contraction	290 (58.00)
	Essential for human fertility	52 (10.40)
	An essential mineral for the synthesis of red blood cells	82 (16.40)
	Helps keep blood sugar in the normal range	14 (2.80)
	Don't know	62 (12.40)

Which mineral is important for maintaining a healthy immune system?	Zinc	285 (57.00)
	Calcium	82 (16.40)
	Potassium	50 (10.00)
	Don’t know	83 (16.60)
Which mineral may cause abnormal heart function in a situation of excessive consumption?	Chromium	77 (15.40)
	Iron	87 (17.40)
	Potassium	163 (32.60)
	Zinc	40 (8.00)
	Don’t know	133 (26.60)
Which main vitamins are essential for maintaining normal bone mass?	Vitamin A, E, and Calcium	74 (14.80)
	Vitamin A, B, and Calcium	58 (11.60)
	Vitamin D, K, and Calcium	269 (53.80)
	Iron, Folic acid, and Hemoglobin	31 (6.20)
	Don’t know	68 (13.60)
Which population will benefit from the benefits of consuming protein beyond the dietary recommendations of 0.8–1 g of protein per kg of body weight for the general population?	Old people suffering from sarcopenia	142 (28.40)
	Chronic kidney failure patients	83 (16.60)
	Young and healthy untrained population	94 (18.80)
	Patients suffering from irritable bowel syndrome	17 (3.4)
	Don’t know	164 (32.80)
What percentage of unplanned weight loss in a period of 3–6 months constitutes a marker that patients are suspected of nutritional deterioration and should be referred to a specialist?	Sudden weight loss of 2.5%	97 (19.40)
	Sudden weight loss of 5%	152 (30.40)
	Sudden weight loss of 1.8%	41 (8.20)
	Sudden weight loss of 3.5%	55 (11.00)
	Don’t know	155 (31.00)
What is the serum albumin index that signals that patients are suspected of being malnourished and/or are receiving a diet with	Less than 5 g/dL	71 (14.20)
	Less than 7.5 g/dL	75 (15.00)
	Less than 4 g/dL	43 (8.60)
	Less than 3.5 g/dL	72 (14.40)

insufficient protein and energy levels, and should be referred to a specialist?	Don't know	239 (47.80)
What are fasting glucose and glycated hemoglobin levels that indicate a diagnosis of diabetes and require a doctor's visit?	Over 126 mg of glucose and 6.5% glycated hemoglobin	173 (34.60)
	Over 100 mg of glucose and 5% glycated hemoglobin	87 (17.40)
	Over 80 mg of glucose and 2.5% glycated hemoglobin	62 (12.40)
	Over 115 mg of glucose and 5.2% glycated hemoglobin	32 (6.40)
	Don't know	146 (29.20)
What are the cholesterol levels that indicate that patients are in dyslipidemia (disorder in the metabolism of blood lipids) and need a referral to nutritional change as part of multi-professional rehabilitation to maintain health?	HDL < 80, LDL > 70, Total cholesterol > 100	98 (19.60)
	HDL < 60, LDL >100, Total cholesterol> 150	72 (14.40)
	HDL < 40, LDL >130, Total cholesterol > 200	98 (19.60)
	HDL < 50, LDL >120, Total cholesterol > 170	31 (6.20)
	Don't know	201 (40.20)
What is the range of values that indicates a danger of morbidity due to being overweight/underweight according to the BMI index?	Between 25–29.9	90 (18.00)
	Between 20–24.9	82 (16.40)
	From 30 and above, and from 18.5 and below	150 (30.00)
	Between 19–29	31 (6.20)
	Don't know	147 (29.40)
What are the identifiable characteristics in patients that indicate that they are at risk of malnutrition and should be referred for further dietary treatment?	Baldness, profuse sweating, accelerated heart rate at rest, profuse secretions, decreased sleep quality, and decreased libido	98 (19.60)

	Functional decline, decrease in muscle mass, loss of subcutaneous fat, unplanned weight loss, and insufficient energy supply through food	179 (35.80)
	Decreased libido, poor appetite, fertility problems, poor sleep, diarrhea, and constipation	34 (6.80)
	Lack of motivation to engage in physical activity, depression, lack of proportion between height and weight, excessive sleep, and low caloric expenditure	41 (8.20)
	Don’t know	148 (29.60)
Mean total nutrition knowledge score (out of 100)	59.68 (17.25)	
